# Panic Disorder as Unthinkable Emotions: Alexithymia in Panic Disorder, a Croatian Cross-Sectional Study

**DOI:** 10.3389/fpsyt.2020.00466

**Published:** 2020-05-20

**Authors:** Daniela Šago, Goran Babić, Žarko Bajić, Igor Filipčić

**Affiliations:** ^1^Day Hospital for Psychotic Disorder, Psychiatric Hospital Sveti Ivan, Zagreb, Croatia; ^2^Faculty of Medicine, Josip Juraj Strossmayer University of Osijek, Osijek, Croatia; ^3^Independent Researcher, Zagreb, Croatia; ^4^School of Medicine, University of Zagreb, Zagreb, Croatia; ^5^Faculty of Dental Medicine and Health, Josip Juraj Strossmayer University of Osijek, Osijek, Croatia

**Keywords:** alexithymia, anxiety disorders, mentalizing, panic disorder, psychoanalytical interpretation

## Abstract

**Objectives:**

Previous research on alexithymia has led to controversy over its prevalence in panic disorder. The aim of this study was to assess the difference in the prevalence of alexithymia in panic disorder and other anxiety disorders.

**Design and Methods:**

We performed a cross-sectional study on a sample of 71 patients diagnosed with panic disorder and 113 patients diagnosed with other anxiety disorders; both groups were 18–50 years old. Primary outcome was the 20-item Toronto Alexithymia Scale (TAS) score. Secondary outcome was the prevalence of alexithymia defined as a TAS score ≥61.

**Results:**

Patients diagnosed with panic disorder had a 25% higher score on the TAS subscale of difficulty identifying feelings than patients diagnosed with other anxiety disorders. The prevalence of alexithymia was 27% in patients with panic disorder and 13% in patients with other anxiety disorders. Patients diagnosed with panic disorder had significantly higher odds for alexithymia.

**Conclusions:**

The results of our study support the hypothesis of higher prevalence of alexithymia in individuals with panic disorder than in individuals with other anxiety disorders. In addition, difficulty identifying feelings as a salient feature of alexithymia is higher in panic disorder than in other anxiety disorders.

## Introduction

The construct of alexithymia refers to a cluster of features, comprising difficulty identifying and describing subjective feelings, an impoverished fantasy life, and preoccupation with external events rather than to inner mental processes (1–3). Initially, alexithymia was described in psychosomatic patients (4–7). As alexithymia theory advanced, the construct progressed beyond the psychosomatic field. For almost half a century, researchers and clinicians argued whether alexithymia is specific to certain groups of patients or not specific and theoretically accessible to all of us, a prerequisite of symptom formation or itself a symptom, primary or secondary, innate or acquired, a state or trait, a defense mechanism, or a structure deficit (3, 8). Nowadays, alexithymia as unique personality trait is considered to reflect deficits in cognitive-emotional processing and regulation of affect (9, 10). While the accurate meaning of the term alexithymia suggests a type of anomia instead of agnosia, for most clinicians and researchers inspired by Sifneos and Nemiah, it is the name of a multifaceted construct that encompasses more than a difficulty finding words for emotional feelings, rather words denuded of their affective significance (11, 12). Alexithymia is also viewed as a massive defense against intolerable emotions as well as a deficit in the mental representation of emotions (9, 12). Alexithymia is in neither the Diagnostic and Statistical Manual of Mental Disorders (DSM) nor the International Classification of Diseases (ICD) diagnostic category. Prevalence of alexithymia is around 10% in the general population (13–17).

Panic disorder (PD) is characterized by recurrent and unexpected panic attacks (18, 19). The lifetime prevalence of PD is approximately 4% (20). PD has high rates of relapse, which suggests a gap in our understanding of the maintain factors behind PD symptoms and that improving our understanding of the psychological processes underlying PD may enhance the efficacy of treatment (21). “Other anxiety disorders” (AODs; ICD-10 F41), including PD, are disorders in which manifestation of anxiety is the major symptom manifested by the somatic and affective component. Somatic arousal activates psychic elaboration whereby metabolize emotions into feelings (22, 23). Analogous to PD, alexithymic persons are prone to misinterpret somatic sensation as signs of physical illness and focus on the somatic manifestation of emotional arousal while minimizing the affective components of emotion (24, 25).

Nemiah was the first author who highlighted the connection between alexithymia and PD (26). In 1993, the first empirical studies were published to confirm this theoretical assumption (27, 28). Subsequently, using the Toronto Alexithymia Scale with 26 item (TAS-26), Bach et al. found no significant association between alexithymia and personality disorders (29). Since 1995, the 20-item Toronto Alexithymia Scale (TAS-20) has been used predominantly for research. The most researchers investigated the prevalence of alexithymia in PD, or in comparison with its prevalence in social phobia, affective disorders, obsessive compulsive disorder, suicidality, personality traits, eating disorders, substance use disorders, and childhood trauma (19, 30–36). De Berardis made a major contribution in this area by studying the impact of alexithymia on anxiety disorders (37, 38). At the same time, theories of pathophysiological mechanisms underlying PD have been developed. Nevertheless, the treatment of PD is still unsatisfactory (39–42). Moreover, alexithymic persons are thought to have poor response to treatment (43, 44). Consequently, there are still many questions to be answered.

In individuals with a normally functioning affective system, somatic arousal activates psychic elaboration (45). Panic can be understood as a core basic signal that is not adequately processed in its significance as a signal function and is not clearly defined or mentalized (46). Elaboration of affect includes identification of the meaning of panic, converting it to an anxiety signal that does not overwhelm the cognitive system (46). It is to be assumed that patients with PD are typically unable to identify bodily experiences and symptoms as representations or symbols of affective states. Somatic symptoms of the PD apparently have neither biological sense nor symbolic significance. The threat is experienced as if it is occurring in the body rather than in the mind, and as if it is a catastrophic danger to the body. This subjective experience indicates a deficit in symbolizing, a sub-symbolic state that has not been represented (46). Bypassing the psyche, anxiety directly affects the soma (47).

We assume that intense anxiety in PD and alexithymia due to insufficient psychological elaboration precipitate into fear and relocates to a somatic symptom (19). This implies that the degree of anxiety is more pronounced in PD than in OAD, leading to a breakdown of the mentalization process. Panic attack as alexithymia is an inability to master bodily arousal and a failure to metabolize primary somatic and affective experiences: a failure to metabolize emotions into feelings (23, 48). PD provides a useful model for exploring maladaptive alarm systems (46). This motivated us to deepen our research on alexithymia in PD.

Contemporary conceptualization of alexithymia inclined toward a dimensional rather than a categorical approach as well as it often included trait and state components (49–53). Nevertheless, the largest number of studies used a categorical model. In line with different methodology, sample size, version of the TAS scale, history of illness, comorbidity, and pharmacological treatment, a diverse range, from 16% to 67%, of alexithymia in individuals with PD was obtained (27, 28, 30, 31, 34, 36, 54–56).

So far researchers have studied alexithymia in Neurotic, stress-related and somatoform disorders. Regarding anxiety disorders, most researches compared alexithymia in PD and phobic disorders where anxiety is evoked only in certain well-defined situations. Since the emotion of anxiety in phobic disorder can be identified and mentalized and is oriented toward external object, it is questionable whether it can be named anxiety or fear.

Considering the above mentioned, our intention was to focus on insufficiently researched diagnostic category, “Other anxiety disorders” (F41). Although this category might seem heterogeneous, the disorders encompassed in it have a common core symptom—a free-floating, unrepresented, unmentalized anxiety directed toward own bodily sensations rather than an external object (like in phobias), which is especially seen in PD and generalized anxiety disorder. While the label “Other anxiety disorders” might imply all anxiety disorders, actually it doesn't encompass F40 codes, under the label of “Phobic anxiety disorders.”

The aim of our study was to assess the prevalence of alexithymia in PD, the difference in alexithymia prevalence in PD and OAD, and correlation between alexithymia and the severity of PD. We hypothesized that the prevalence of alexithymia is higher in patients with PD (F41.0) than in patients with OAD (F41.1, F41.2, F41.3, F41.8, and F41.9). According to our knowledge, this is the first study comparing these diagnostic categories. We also bring into focus the qualitative assessment between PD and alexithymia, using the Panic Disorder Severity Scale (PDSS).

## Methods

### Study Design

Cross-sectional study was performed during three years in Mental Health Center Zagreb, Croatia. The study protocol was approved by the Ethics Committee of the Mental Health Center Zagreb. All participants provided written informed consent for participation, and their identities were concealed by assigning them a numerical code. We kept the signed informed consent forms and completed questionnaires separate. Informed consent included the aim and the purpose of the research. The study was performed in accordance with the World Medical Association Declaration of Helsinki 2013 (57).

### Subjects

Our targeted population was outpatients newly diagnosed with PD (ICD-10 F41.0). The control population was patients newly diagnosed with OAD (ICD-10 F41.1–F41.9). We selected a consecutive sample, enrolling the patients by the order of their arrival to the first psychiatric evaluation. Inclusion criteria included both genders, age 18–50 years, and ability to complete the questionnaires by themselves. Exclusion criteria consisted of presence of other psychiatric or somatic disorders, previous PD or OAD, psychotherapy or pharmacotherapy, and acute suicidality. Of the 192 respondents, two did not give their consent to participate in the study, five had psychiatric or somatic comorbidity, and one had a previous psychotherapy so they were excluded from the sample. Therefore, 71 patients with PD and 113 with OAD were included in the study.

The diagnosis was made by an experienced psychiatrist during a clinical interview according to the ICD-10 criteria verified by the PDSS for patients with PD. The PDSS was administered by the same psychiatrist. Participants were screened for exclusion criteria during the clinical interview, and in the self-report questionnaire (made for the purpose of this research) that included items regarding comorbidities and previous pharmacological and psychotherapy treatment. We performed the power analysis before the enrollment. We obtained the expected TAS-20 values from the Cucchi et al. study (35), set the needed power at 80%, the significance at p <0.05, and the minimal difference at least as large as the one found in the Cucchi et al. study. The largest sample size was needed for the externally oriented thinking (EOT) TAS-20 subscale, n = 58 in each group. We expected up to 10% of incorrectly collected data and determined the initially needed sample size to be 65 in each group. The needed sample size was calculated using PASS 13 Power Analysis and Sample Size Software (2014) (NCSS, LLC; Kaysville, Utah, USA, ncss.com/software/pass).

### Outcomes

Our primary outcome was the 20-item TAS-20 score (58, 59). Each item was rated on a five-point Likert-type scale, ranging from “strongly disagree” (scored 1) to “strongly agree” (scored 5), with scores ranging from 20 to 100. Higher total scores indicated more alexithymia. TAS-20 has three subscales that assess difficulty identifying feelings (DIF), difficulty describing feelings (DDF) to others, and EOT. The scale has been translated into numerous languages, and its three-factor structure, has been cross-validated by confirmatory factor analysis across many countries and cultures (60–65). These findings support the view that alexithymia is a common trait rather than a culture-specific construct (3). Our secondary outcome was the prevalence of alexithymia. TAS-20 at the same time provides continuous assessment of alexithymia characteristics to identify persons with high and low alexithymia as well as empirically derived cutoff scores (58). Subjects with a total score of ≤51 are considered non-alexithymic, those scoring 52 to 60 possibly alexithymic, and subjects scoring ≥61 considered alexithymic (66). As a tertiary, exploratory outcome, we analyzed the association of the TAS-20 score with the severity of PD measured by the PDSS (67, 68).

The PDSS was developed to provide a simple method of measuring the overall severity of PD (68). It consists of seven items, each rated on a five-point Likert scale. The items consider panic frequency, distress during panic, anticipatory anxiety, phobic avoidance of situations, phobic avoidance of physical sensations, impairment in work functioning, and impairment in social functioning (68). A total score is calculated by summing the scores for all seven items. Individual responses are scored on a scale of 0–4, and total scores range from 0 to 28.

### Possible Confounders

Possible confounders of our primary and secondary outcomes whose effects we tried to control by the multivariable statistical analysis were: age in years, gender, education, partnership, having children, work status, and number of household members. All this data was collected by participants' self-reports by answering the questionnaires. We did not independently check the validity of any of this data.

### Statistical Analysis

In the analysis of our primary outcome, we calculated the absolute difference between medians of the TAS-20 and its three subscales in PD and OAD patients with Bonett–Price 95% confidence intervals (CI), the difference relative to the median in the OAD group; the statistical significance of the difference, using quantile regression; and Cliff's delta as the standardized effect size in the crude, unadjusted, and in the analysis adjusted for all preplanned possible confounding factors. We used the sequential Holm–Bonferroni correction to control the false-positive rate caused by multiple testing. In the analysis of our secondary outcome, we analyzed the difference in the prevalence of alexithymia in PD and OAD by multivariable, adjusted, binary logistic regression. Finally, we analyzed the unadjusted correlation between the TAS-20 score and the severity of PD measured by the PDSS in the PD group, using Kendall's tau b coefficient, and the independent correlation after the adjustment for preplanned possible confounding factors, using a quantile regression. We set the level of significance at two-tailed p <0.05 and all CIs at 95%. We performed statistical data analysis by using the R Core Team (2018). R is a language and environment for statistical computing (R Foundation for Statistical Computing; Vienna, Austria; URL https://www.R-project.org).

The data that support the findings of this study are available from the corresponding author, upon reasonable request.

## Results

We enrolled 71 patients diagnosed with PD and 113 patients diagnosed with OAD. Two study groups were balanced regarding the gender, age, education, partnership status, having children, work status, and number of household members (**Table 1**).

**Table 1:** Participants**'** sociodemographic characteristics.

	Panic disorder (n = 71)		Other anxiety disorder (n = 113)	
Sociodemographic characteristics				
Gender				
Men	29	(40.8)	45	(39.8)
Women	42	(59.2)	68	(60.2)
Age (years), median (IQR)	29	(24–36)	32	(23–36)
Education				
High school	49	(69.0)	70	(61.9)
University	22	(31.0)	43	(38.1)
Partnership				
Single	18	(25.4)	30	(26.5)
In a steady partnership	13	(18.3)	26	(23.0)
Married	40	(56.3)	57	(50.4)
Having children				
Yes	40	(56.3)	61	(54.5)
No	31	(43.7)	51	(45.5)
Work status				
Employed or student	47	(66.2)	82	(73.2)
Unemployed or retired	24	(33.8)	30	(26.8)
Number of household members, median (IQR)	3	(3–4)	4	(3–4)
Number of household members				
≤2	17	(23.9)	24	(21.2)
3	20	(28.2)	31	(27.4)
4	27	(38.0)	35	(31.0)
≥5	7	(9.9)	23	(20.4)

Data is presented as number (percentage) of participants if not stated otherwise.

IQR, interquartile range.

Data was missing for working status for one (0.9%) patient; with other anxiety disorder in one (1.4%) patients with panic disorder.

After the adjustment for preplanned possible confounding factors and sequential Holm–Bonferroni correction for multiple testing, patients diagnosed with PD had significantly higher scores on the DIF subscale (**Table 2**). The difference in adjusted medians was Δ = 4 (95% CI: 3.1–5.6), which was 25% relative to the score in the OAD group. This was a moderately high standardized effect size of Cliff's δ = 0.61 (95% CI: 0.48–0.70). The differences between PD and OAD patients regarding the other two TAS-20 subscales, DDF and EOT, as well as the total TAS-20 score, were not significant.

**Table 2:** Toronto Alexithymia Scale (TAS-20) results in two study groups.

	Panic disorder (n = 71)		Other anxiety disorder (n = 113)		Δ	(95% CI)	Δ%	δ	(95% CI)	p	p_corr_
	Median	(IQR)	Median	(IQR)							
Crude, unadjusted											
TAS total score	53	(43–63)	46	(37–54)	7	(2.0–12.0)	15%	0.25	(0.08–0.41)	0.004	0.024
TAS subscales											
DDF	13	(10–16)	12	(9–15)	1	(−0.8–2.8)	8%	0.17	(−0.01–0.33)	0.261	0.522
DIF	20	(14–26)	15	(12–21)	5	(2.9–7.1)	33%	0.27	(0.10–0.43)	0.002	0.016
EOT	20	(16–22)	19	(15–21)	1	(−0.8–2.8)	5%	0.09	(0.08–0.25)	0.213	0.639
Adjusted*											
TAS total score (20-100)	52	(51–57)	45	(42–48)	7	(5.9–8.0)	16%	0.66	(0.55–0.74)	0.012	0.060
TAS subscales											
DDF	14	(12–15)	12	(11–13)	2	(1.6–2.4)	17%	0.56	(0.42–0.67)	0.065	0.260
DIF	20	(17–23)	16	(13–18)	4	(3.1–5.6)	25%	0.61	(0.48–0.70)	0.003	0.021
EOT	19	(18–20)	18	(17–20)	1	(0.4–1.7)	6%	0.15	(−0.01–0.30)	0.680	0.680

DDF, difficulty describing feelings; DIF, difficulty identifying feelings; EOT, externally oriented thinking; Δ, absolute median difference; CI, Bonett-Price confidence interval; Δ% median difference relative to the value in other anxiety disorder group; δ, Cliff's delta given as the standardized effect size of the difference in medians; p, statistical significance of the difference between the panic disorder and other anxiety-disorder groups calculated by quantile regression; p_corr_, statistical significance corrected by the sequential Holm–Bonferroni correction for multiple testing.

* Medians were adjusted for age, gender, education, working and partnership status, having children, and number of household members.

In PD, the unadjusted prevalence of alexithymia was 19/71 (27; 95% CI: 17%–39%) and 15/113 (13%, 95% CI: 8%–21%) in OAD. Patients diagnosed with PD had unadjusted odds for alexithymia odds ratio (OR) = 2.39 (95% CI: 1.05–2.28) compared to patients diagnosed with OAD. PD patients had 61% higher relative risk for alexithymia than OAD patients, relative risk (RR) = 1.61 (95% CI: 1.03–2.28). After the adjustment for preplanned possible confounding factors by multivariable binary logistic regression, patients diagnosed with PD had significantly and more than two times higher odds for alexithymia, defined as TAS-20 score ≥61, than patients diagnosed with OAD (OR = 2.75; 95% CI: 1.19–6.36; p = 0.018).

**Figure 1:**
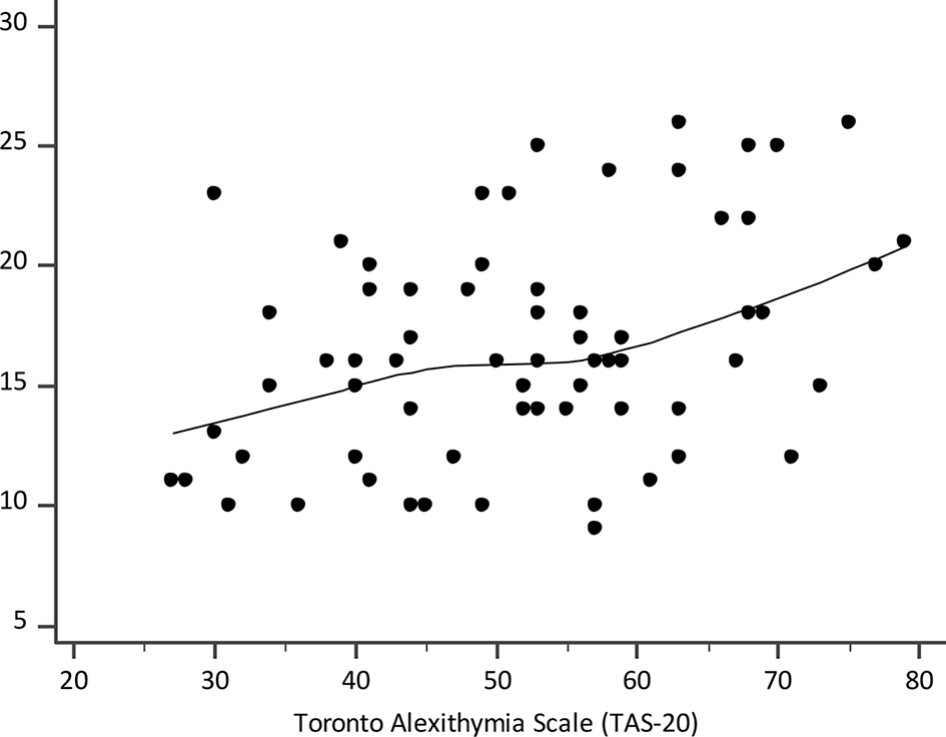
Scatter diagram of correlation between the Toronto Alexithymia Scale (TAS-20) and the Panic Disorder Severity Scale (PDSS); the line is a locally estimated scatterplot smoothing (LOESS) trend-line with the smoothing span of 80%.

In unadjusted analysis in the PD group, the severity of PD measured by the PDSS was significantly correlated with the total TAS-20 score (Kendall's tau b, τ = 0.16, p = 0.011) (**Figure 1**), subscales: DIF (Kendall's tau b, τ = 0.14, p = 0.030), and DDF (Kendall's tau b, τ = 0.17, p = 0.010), but not with the EOT subscale (Kendall's tau b, τ = 0.09, p = 0.186). After the adjustment for preplanned covariates by quantile regression, the total TAS-20 score and none of its subscales were significant independent predictors of the severity of PD (total TAS-20, p = 0.065; DIF, p = 0.187; DDF, p = 0.159; EOT, p = 0.060).

## Discussion

The results of this study are limited and only partially confirm our hypothesis. In this study, TAS-20 DIF subscale scores were significantly higher in the PD group than in the OAD. This is consistent with the results of previous similar research (27, 28, 36, 69). This result supports the hypothesis of a higher rate of alexithymia in patients with PD than in those with OAD, as well as understanding alexithymia as the difficulty to identify emotions. Capacity to communicate and name one's emotion, in other words DIF subscale, is a central aspect in alexithymia (19, 70).

In this survey, the prevalence rate of alexithymia in PD was 27%. The first two studies of alexithymia and PD that used the TAS-26 suggested that alexithymia may constitute one of the psychological characteristics of PD, with a prevalence of 67% and 46.7%, respectively (27, 28). The first and highest prevalence rate of alexithymia in PD could be explained due to the relatively small sample of 27 patients (28). Successively, Iancu and colleagues (56) reported a prevalence of alexithymia of 39% by use of the TAS–26, whereas Cox and colleagues (30) found a prevalence of 34.0% with the TAS-20. In a more recent and well-conducted follow-up study on 52 adult patients with PD, Marchesi et al. (32) reported a prevalence for alexithymia of 44.2%. Contrary to De Berardis and colleagues (34), many studies examined PD patients with a relatively long history of illness, and this could lead to potential biases due to actual or previous psychological and pharmacological treatments that may, on their own, influence psychological characteristics, such as alexithymia. We used very selective inclusion criteria and restricted the observation to outpatients with recent-onset PD only, and without a history of previous regular treatment in an attempt to avoid potential biases as secondary alexithymia or somatic symptoms secondary to a psychiatric disorder (34, 71).

The differences between PD and OAD patients regarding DDF and EOT, the other two TAS-20 subscales, and the total TAS-20 score, as well as PDSS were not significant.

Most researchers agree that DIF and DDF are salient features of alexithymia, although some researchers have suggested that the EOT subscale is substantially different from the DIF and DDF subscales (72, 73). In our study, the EOT subscale appears to be a quite independent variable from PD, and this finding resonates with those of previous studies. Many authors emphasize the difference and caution interpreting of the EOT subscale scores (74, 75). The EOT dimension corresponds closely to a concept of pensée opératoire (operative thinking) introduced by French researchers Marty and de M'Uzan. An operative or utilitarian style of thinking includes the absence of fantasy and other manifestations of the depleted inner mental world of feelings and ideas about intentions, needs, and attitudes and focused on external events (3, 6). Whereas healthy individual integrate dreams, fantasies, or symbolic interactions in symbolic representations, the alexithymic individual fills his or her inner world with external details, a fact that can be particularly well observed (6, 8). The origin of alexithymia can be located at a stage prior to the formation of representations (8). The failure in the mother–child symbiosis prevents the development of symbolic thinking (8). Emptiness instead of good object, actions instead of language direct person from inside to outside reality, to concrete presence of external objects instead of fantasy and internalization (8, 47). The disturbances of the mother's alfa function do not signalize so much of separation anxiety, but rather an unspecified fear of annihilation (8, 46, 76). No good object representations are accessible, and such individuals remain reliant on on the concrete presence of external objects (8, 47). Object loss represents one of two dangerous extremes of “oneness to none-ness” and precipitates the formation of somatic symptoms (8).

In terms of affective states, panic patients have particular difficulty identifying, verbalizing, and representing certain affects, although this capacity can vary depending on the context (46). The experience of certain emotions, including anger, dependent feelings, and separation fears, as dangerous is relevant to this difficulty, but identification of these feelings is necessary to identify the danger. In fact, understanding that emotions can trigger anxiety can be seen as part of the process of representing affects. Mentalization as capacity to understand ourselves and others could enhance alexithymic characteristics particularly items in DIF subscale which were negatively correlated with bias in emotion perception, especially in social relation (77). Exploration and interpretation of avoided emotions improve reflective function and mentalization in PD (78).

We suppose that patients with PD, unlike OAD, suffer from the lack of an integrated representational system that links affective, somatic, and verbal realms, adding to the vulnerability to panic and severe anxiety onset and persistence. These bodily and emotional experiences are dissociated from meaningful conscious links and the verbal symbolic realm. Psychoanalytical interpretation enables us to understand alexithymia and PD as an insufficiency in the process of mentalization and, consequently, a deficiency in the regulation of affect.

### Limitations of the Study

This study has several limitations. Among the major one is quite dispersive and heterogeneous OAD as a comparison group. In addition to “Generalized Anxiety Disorder” (F41.1), “Other specified anxiety disorders” (F41.8), and “Anxiety disorder, unspecified” (F41.9), other diagnoses such as “Mixed anxiety and depressive disorder” (F41. 2), and “Other mixed anxiety disorders” (F41.3) may contain other symptoms, in particular depressive symptoms, that may affect the results.

The data were collected at one site and by only one rater, experienced psychiatrist. The advantage of such approach is homogeneity, but it could also lead to reduced possibility of generalization as well as lead to systematic error.

The data were mostly based on self-report. This method has some inherent flaws such as poor self-awareness, an increased risk for socially desirable answers, and various response styles (79, 80). Measuring alexithymia by TAS-20 has some shortcomings even though captures an impairment in feeling and describing emotions and is a valid measure of the alexithymia construct (12). The concurrent use of the Toronto Structured Interview for Alexithymia (TSIA) with TAS-20 may overcome some of these limitation (81).

We used a cross-sectional design that is limited in terms of causality.

Concerning theoretical part, there is an additional significant limitation. Psychodynamic theory did not emerge as a result of this study, even though it makes it easier to understand that anxiety in PD is overwhelming and different from anxiety in OAD. Considering the global scientific literature the biological theory of PD are well-researched yet poorly understood while psychodynamic thought is scarcely validated by empirical evidence-based research (82–85). Fostering a multi-perspective approach to PD affords distinctive glimpses into psychopathology and enhances our understanding of PD (86).

## Conclusions

Even though anxiety is the core symptom of the ICD-10 F41 (F41.0–F41.9) diagnostic category, there is a difference between PD (F41.0) and OAD (F41.1–F41.9). The construct of alexithymia enable us to think of PD as an unthinkable emotion. Alexithymia and the “unmentalized” psychosomatic mind in some way distinguish PD from OAD. Alexithymia represents difficulties in affective and cognitive differentiation of emotions characterized by poor verbalization, resomatization, and dedifferentiation of mental and somatic affective experience; similarly, PD is a perfect example of such dedifferentiation and resomatization of affects in undifferentiated form.

Elevated rates of alexithymia in PD may reflect patients' inclination to constrict their emotional experience to avoid affect-based psychological sensation (56). Individuals with alexithymic features, as well as individuals with PD, are unable to use bodily perceptions as a helpful signal for themselves. Enhancing or improving symbolic thinking or mentalizing might be beneficial for these individuals.

## Data Availability Statement

The datasets generated for this study are available on request to the corresponding author.

## Ethics Statement

The studies involving human participants were reviewed and approved by Ethics Committee of the Health Center Zagreb. The patients/participants provided their written informed consent to participate in this study.

## Author Contributions

All authors contributed substantially to the conception and design, reviewed the manuscript, and approved the submitted version.

## Conflict of Interest

The authors declare that the research was conducted in the absence of any commercial or financial relationships that could be construed as a potential conflict of interest.
